# Impact of Soluble CD26 on Treatment Outcome and Hepatitis C Virus-Specific T Cells in Chronic Hepatitis C Virus Genotype 1 Infection

**DOI:** 10.1371/journal.pone.0056991

**Published:** 2013-02-20

**Authors:** Jonas Söderholm, Jesper Waldenström, Galia Askarieh, Massimo Pilli, Pierre-Yves Bochud, Francesco Negro, Jean-Michel Pawlotsky, Stefan Zeuzem, Carlo Ferrari, Gunnar Norkrans, Rune Wejstål, Johan Westin, Avidan U. Neumann, Bart L. Haagmans, Magnus Lindh, Gabriele Missale, Kristoffer Hellstrand, Martin Lagging

**Affiliations:** 1 Department of Infectious Diseases, University of Gothenburg, Gothenburg, Sweden; 2 Azienda Ospedaliera di Parma, Parma, Italy; 3 Service of Infectious Diseases, Department of Medicine, University Hospital and University of Lausanne, Lausanne, Switzerland; 4 Hospital University of Geneva, Geneva, Switzerland; 5 Hôpital Henri Mondor - Université Paris XII, Créteil, France; 6 INSERM U955, Créteil, France; 7 Department of Medicine I, J. W. Goethe University Hospital, Frankfurt, Germany; 8 Bar-Ilan University, Ramat-Gan, Israel; 9 Department of Virology, Erasmus MC, Rotterdam, The Netherlands; Copenhagen University Hospital, Denmark

## Abstract

**Background:**

Interferon and ribavirin therapy for chronic hepatitis C virus (HCV) infection yields sustained virological response (SVR) rates of 50–80%. Several factors such as non-1 genotype, beneficial *IL28B* genetic variants, low baseline IP-10, and the functionality of HCV-specific T cells predict SVR. With the pending introduction of new therapies for HCV entailing very rapid clearance of plasma HCV RNA, the importance of baseline biomarkers likely will increase in order to tailor therapy. CD26 (DPPIV) truncates the chemokine IP-10 into a shorter antagonistic form, and this truncation of IP-10 has been suggested to influence treatment outcome in patients with chronic HCV infection patients. In addition, previous reports have shown CD26 to be a co-stimulator for T cells. The aim of the present study was to assess the utility of CD26 as a biomarker for treatment outcome in chronic hepatitis C and to define its association with HCV-specific T cells.

**Methods:**

Baseline plasma from 153 genotype 1 and 58 genotype 2/3 infected patients enrolled in an international multicenter phase III trial (DITTO-HCV) and 36 genotype 1 infected patients participating in a Swedish trial (TTG1) were evaluated regarding baseline soluble CD26 (sCD26) and the functionality of HCV-specific CD8^+^ T cells.

**Results:**

Genotype 1 infected patients achieving SVR in the DITTO (*P = *0.002) and the TTG1 (*P* = 0.02) studies had lower pretreatment sCD26 concentrations compared with non-SVR patients. Sixty-five percent of patients with sCD26 concentrations below 600 ng/mL achieved SVR compared with 39% of the patients with sCD26 exceeding 600 ng/mL (*P* = 0.01). Patients with sCD26 concentrations below 600 ng/mL had significantly higher frequencies of HCV-specific CD8^+^ T cells (*P = *0.02).

**Conclusions:**

Low baseline systemic concentrations of sCD26 predict favorable treatment outcome in chronic HCV infection and may be associated with higher blood counts of HCV-specific CD8^+^ T cells.

## Introduction

Until recently, the standard-of-care therapy for patients chronically infected with hepatitis C virus (HCV) has been a combination of pegylated interferon-α (pegIFN-α) and ribavirin. This treatment results in sustained virological response (SVR) rates of 50–80% [1]. In addition to HCV non-1 genotypes, factors such as lower baseline HCV RNA levels, lower body mass index (BMI), younger age, female gender, lower alanine transaminase (ALT), less advanced liver fibrosis, and beneficial *IL28B* single nucleotide polymorphisms (SNPs) are associated with a favorable treatment response [2]–[6]. Additionally, the newly approved HCV protease inhibitors have entailed significantly improved outcome for HCV genotype 1 infected patients [7], [8]. With the pending introduction of newer Direct-Acting Antiviral (DAA) agents, *e.g.* nucleotide NS5B inhibitors, which yield very rapid initial clearance of plasma HCV RNA but yet sizeable relapse rates [9], especially in difficult-to-cure genotype 1a infected patients, the importance of baseline biomarkers likely will increase in order to tailor choice of therapy and treatment duration.

High systemic and intra-hepatic levels of the IFN-γ-inducible protein 10 kDa (IP-10 or CXCL10) predict treatment failure following pegIFN-α-based therapy in chronic HCV infection [10]–[15]. In addition, reports have shown both IP-10 and the *IL28B* SNP variations to be associated with the antiviral response to pegIFN-α/ribavirin therapy [10], [13], [16]. IP-10 attracts T cells, NK cells, and monocytes to the site of infection via the CXC chemokine receptor 3 (CXCR3) [17]. IP-10 is degraded into an antagonistic form by the dipeptidyl peptidase enzymatic activity of CD26 (also known as DPPIV), a serine protease that cleaves a dipeptide from the N-terminus of polypeptides with either a proline or alanine at the penultimate position [18], [19]. A recent study by Casrouge *et al.* reported that patients failing therapy have higher baseline plasma DPPIV activity [20]. The authors suggested that a subsequent systemic accumulation of the truncated antagonistic form of IP-10 impairs migration of CXCR3^+^ cells to the infected liver. CD26 is present both as a membrane-bound and as a soluble (sCD26) form, and the DPPIV activity of the membrane-bound form has been suggested to be important for T cell activation [21] possibly by associating with CD45 [22]. Also, sCD26 has been shown to enhance CD86 expression on antigen-presenting cells [23].

Dysfunctional exhausted HCV-specific T cells have been associated both with the establishment of HCV chronicity [24], [25] and with treatment failure following pegIFN-α-based therapy [26]. In line with these findings, functional HCV-specific T cell responses were observed in patients spontaneously resolving acute HCV infection [27] and in chronically infected patients achieving SVR following treatment [28]. The aim of the present study was to evaluate the impact of sCD26 on the outcome of HCV therapy and its association with the functionality of HCV-specific T cells.

## Methods

### Ethical Aspects

The treatment studies conformed to the guidelines of the 1975 Declaration of Helsinki and were approved by ethical committees at each center (Medicinska fakultetens forskningsetikkommitté, Göteborgs Universitet, Gothenburg, Sweden, CPP-Ile-de-France IX, CHU Henri Mondor, Creteil, France, Comitato Etico Indipendente (IRB/IEC) dell'Azienda Ospedaliera di Parma, Parma, Italy, Comite d’Ethique du department de Medicine, Hopitaux Universitaires de Genève, Geneva, Switzerland, the Helsinki committee of the Kaplan Medical Center, Rehovot, Israel, the Ethics Committee of Hospital General Vall d’Hebron, Barcelona, Spain, the Ethics Committee of Aristotle University of Thessaloniki, Thessaloniki, Greece, the Ethics Committee of Klinikum der Johann Wolfgang Goethe-Universitat, Frankfurt, Germany, and the Ethics Committee of University Hospital Rotterdam Dijkzigt, Rotterdam, Netherlands). Written informed consent was obtained from each participating patient.

### Patients

Between February 2001 and November 2003, 270 patients were recruited in a phase III, open-label, randomized, multicenter trial conducted by the DITTO-HCV study group at nine centers in France, Germany, Greece, Israel, Italy, Netherlands, Spain, Sweden, and Switzerland, as previously reported [29]. All patients were adults, had compensated liver disease, were treatment-naïve for hepatitis C, and fulfilled the following inclusion criteria: a positive test for anti-HCV antibody, baseline HCV RNA above 1000 IU/mL, and two serum alanine aminotransferase values above the upper limit of normal within 6 months of treatment initiation. The present study included the 211 of the initial 250 genotype 1–3 patients in the DITTO study where previously unthawed plasma samples were available. Baseline plasma samples for 153 genotype 1 (Table 1) and 58 genotype 2/3 (Table 2) patients were analyzed for sCD26 concentrations, and all of them had previously been analyzed for both *IL28B* genetic variants [13], [16] and baseline plasma IP-10 [14], [15]. Twenty-eight human leukocyte antigen (HLA)-A2 and HLA-A3 positive (Table 1 and 2) patients chosen based on the availability of sufficient numbers of viable peripheral blood mononuclear cells (PBMCs) were studied for HCV-specific HLA class I responses before treatment initiation, as previously described [28]. The plasma samples were stored in aliquots at −80°C and the PBMCs were stored in liquid nitrogen until assayed.

**Table 1:** Baseline and on-treatment characteristics of the genotype 1 patients in the complete DITTO study, the genotype 1 patients available for the present study, and the genotype 1 patients available for the T cell study.

	Complete genotype 1 DITTO cohort (n = 173)	c *P*	Genotype 1 patientsincluded (n = 153)	d *P*	Genotype 1 T cellstudy patients (n = 24)
**Log_10_ HCV RNA (IU/mL) decline** **1^st^ day on treatment**	0.67 (−1.05–3.07)	1.0^a^	0.67 (−1.05–3.07)	0.5^a^	0.62 (0.24–2.46)
**Log_10_ HCV RNA (IU/mL) decline** **day 8 to 29 on treatment**	1.48 (−2.94–5.81)	0.8^a^	1.39 (−2.94–5.81)	0.1^a^	0.89 (0.36–4.52)
**RVR**	38 (22%)	0.8^b^	35 (23%)	0.8^b^	5 (21%)
**SVR**	93 (54%)	0.9^b^	81 (53%)	0.8^b^	12 (50%)
**Female**	52 (30%)	0.9^b^	47 (31%)	0.5^b^	9 (38%)
**BMI (kg/m^2^)**	24.6 (18.8–39.9)	1.0^a^	24.4 (18.8–39.8)	0.2^a^	26.0 (11.5–20.3)
**Age (years)**	43.0 (18.0–64.0)	1.0^a^	42.0 (18.0–64.0)	0.2^a^	44.0 (30.0–64.0)
**IP-10 (pg/mL)**	240 (29.0–2244)	1.0^a^	240 (29.0–2200)	0.6^a^	286 (49–706)
**IP-10 (<150/150–600/>600 pg/mL)**	47/102/24	1.0^b^	42/89/22	0.5^b^	5/17/2
**sCD26 (ng/mL)**	n.a.	n.a.	588 (66–1696)	0.3^a^	525 (187–1013)
**DPPIV (AU)**	n.a.	n.a.	31.6 (15.6–113.1)	0.2^a^	37.7 (18.0–113.0)
**HCV RNA (IU/mL)**	6.27 (2.92–7.38)	0.7^a^	6.19 (2.92–7.22)	0.7^a^	6.35 (4.16–7.21)
***rs12979860*** ** (CC/CT/TT)**	46/98/29	1.0^b^	40/88/25	0.3^b^	6/17/1
***rs8099917*** ** (TT/TG/GG)**	99/67/7	0.9^b^	85/61/7	0.9^b^	12/11/1
***rs12980275*** ** (AA/AG/GG)**	54/90/29	1.0^b^	47/81/25	0.2^b^	7/16/1
**ALT (IU/L)**	50.5 (18.0–335.3)	0.9^a^	56.0 (18.0–335.3)	0.7^a^	76.9 (25.0–224.0)
**Fibrosis Stage (Ishak 0/1/2/3/4/5/6)**	11/46/40/20/10/12/10	1.0^b^	10/40/35/15/8/12/10	0.8^b^	2/4/6/3/1/1/0

Median (range) or individuals (percent). Statistics using^ a^Mann-Whitney *U* test and ^b^χ^2^ test.

c *P*-value for all genotype 1 patients in the complete DITTO study compared with the patients available for sCD26 analysis.

d *P*-value for all genotype 1 patients available for analysis of sCD26 compared with HLA-A2 or HLA-A3 patients available for the tetramer study. n.a. = not applicable.

**Table 2:** Baseline and on-treatment characteristics of the genotype 2/3 patients in the complete DITTO study, the genotype 2/3 patients available for the present study, and the genotype 2/3 patients available for the T cell study.

	Complete genotype 2/3 DITTO cohort (n = 77)	c *P*	Genotype 2/3 patients included (n = 58)	d *P*	Genotype 2/3 T cell study patients (n = 4)
**Log_10_ HCV RNA (IU/mL) decline** **1^st^ day on treatment**	1.83 (−0.66–4.18)	0.8^a^	1.82 (0.12–3.14)	0.3^a^	2.19 (1.02–2.50)
**Log_10_ HCV RNA (IU/mL) decline** **day 8 to 29 on treatment**	2.51 (0.00–4.65)	0.9^a^	2.51 (0.00–4.65)	0.1^a^	1.26 (0.00–3.24)
**RVR**	62 (81%)	0.7^b^	45 (78%)	0.6^b^	4 (100%)
**SVR**	68 (88%)	0.2^b^	55 (95%)	1.0^b^	4 (100%)
**Genotype (2/3)**	24/53	0.5^b^	21/37	0.6^b^	2/2
**Female**	29 (38%)	0.7^b^	24 (41%)	1.0^b^	2 (50%)
**BMI (kg/m^2^)**	24.6 (18.7–32.4)	0.5^a^	25.3 (19.3–32.4)	0.9^a^	24.9 (22.8–29.4)
**Age (years)**	42.0 (20.0–66.0)	0.7^a^	43.0 (20.0–66.0)	1.0^a^	42.5 (36.0–49.0)
**IP-10 (pg/mL)**	188 (43–2200)	0.9^a^	199 (43–1843)	0.04^a^	97 (64–157)
**IP-10 (<150/150–600/>600 pg/mL)**	28/42/7	1.0^b^	21/32/5	0.3^b^	3/1/0
**sCD26 (ng/mL)**	n.a.	n.a.	505 (75–1134)	0.8^b^	493 (389–610)
**DPPIV (AU)**	n.a.	n.a.	32.3 (9.0–60.0)	0.4^b^	28.5 (11.0–54.0)
**HCV RNA (IU/mL)**	6.43 (3.14–7.43)	0.7^a^	6.48 (3.14–7.43)	0.9^a^	6.35 (4.16–7.21)
***rs12979860*** ** (CC/CT/TT)**	50/23/4	0.9^b^	36/18/4	0.2^b^	1/3/0
***rs8099917*** ** (TT/TG/GG)**	57/19/1	0.9^b^	41/16/1	0.6^b^	2/2/0
***rs12980275*** ** (AA/AG/GG)**	50/23/4	0.9^b^	37/17/4	0.2^b^	1/3/0
**ALT (IU/L)**	96.0 (25.0–702.0)	0.8^a^	95.4 (25.0–702.0)	0.6^a^	79.5 (41.3–192.0)
**Fibrosis Stage (Ishak 0/1/2/3/4/5/6)**	0/15/23/10/7/7/4	1.0^b^	0/12/16/9/5/6/2	0.2^b^	0/0/4/0/0/0/0

Median (range) or individuals (percent). Statistics using^ a^Mann-Whitney *U* test and ^b^χ^2^ test.

c *P*-value for all genotype 2/3 patients in the study compared with the patients available for sCD26 analysis.

d *P*-value for all genotype 2/3 patients available for analysis of sCD26 compared with HLA-A2 or HLA-A3 patients available for the tetramer study. n.a. = not applicable.

The second study (the TTG1 trial) was conducted between 2008 and 2010 in 106 Swedish patients chronically infected with genotype 1. For the present study, previously unthawed samples from 36 patients treated at the Sahlgrenska University Hospital, Gothenburg, Sweden were retrieved for analysis. The plasma samples were stored in aliquots at −80°C until assayed.

### Treatment

All patients in the DITTO-HCV trial were initially treated for six weeks with 180 µg pegIFN-α2a administrated by subcutaneous injections once weekly (Pegasys, F. Hoffmann-LaRoche, Basel, Switzerland) and ribavirin orally twice daily (Copegus, F. Hoffmann-LaRoche) at a total daily dose of 1,000 mg for patients weighing less than 75 kg and 1,200 mg daily for above 75 kg. After six weeks of therapy, 50% of the patients were randomized based on their viral kinetic classification to receive individualized therapy or to continue on standard combination therapy for a total of 48 weeks. There were, however, no major differences in treatment outcome for patients receiving individualized or standard therapy [29]. Patients were classified as having SVR if HCV RNA was undetectable in plasma 24 weeks after the completion of therapy and classified as having a rapid virological response (RVR) if HCV RNA was undetectable in plasma week 4 after treatment initiation.

For the TTG1 trial, patients were randomized to receive 180 µg pegIFN-α2a subcutaneous injections once weekly and ribavirin orally twice daily at a total daily dose of 1,000 mg for patients weighing less than 75 kg and 1,200 mg daily for above 75 kg either for the standard-of-care (SOC) response-guided duration or individually tailored treatment duration. The treatment duration in the SOC group was 24, 48, or 72 weeks depending on whether HCV RNA was undetectable after 4, 12, or 24 weeks of treatment, with patients discontinuing if HCV RNA had not declined by at least 2 log_10_ IU/mL after 12 weeks. In the tailored group, there was a flexible treatment duration depending on rate of HCV RNA decline.

### HCV Genotyping

Genotyping of HCV was for the DITTO-HCV trial performed using INNO-LiPA HCV II (Innogenetics N.V., Ghent, Belgium) and for the TTG1 trial using Taqman real-time PCR [30].

### HCV RNA Quantification

HCV RNA was quantified in the DITTO study using Cobas Amplicor (Roche Diagnostics, Branchburg, NJ) on days 0, 1, 4, 7, 8, 15, 22, 29, and weeks 6, 7, 8, 10, 12, 18, 24, 30, 36, 42, 48, 54, as well as 24 weeks after the completion of the treatment. Samples in the TTG1 study were obtained at baseline and after 1, 2, 3, 4, 7, 8, 12, 16, 20, and 24 weeks of treatment.

### Characterization of Single Nucleotide Polymorphisms

The *IL28B*-related SNPs *rs12979860, rs12980275* and *rs8099917* were determined for the DITTO-HCV trial with TaqMan SNP genotyping assays (Applied Biosystems Inc., Foster City, CA) as previously described [13], and the *IL28B*-related SNPs *rs12979860* and *rs8099917* were for the TTG1 trial determined by allelic discrimination using Taqman MGB (minor groove binding) probes [31].

### Analysis of sCD26 Concentration

Concentrations (ng/mL) of soluble CD26/DPPIV, human sCD26 ELISA (BMS235CE, eBioscience, San Diego, CA, USA) were quantified according to the manufacturer’s protocol with the plasma samples diluted 1∶5 in sample diluent [32].

### Analysis of DPPIV Activity

The DPP enzymatic activity of sCD26 was measured using the DPPIV-Glo™ Protease Assay (Promega, Madison, WI, USA), which is based on the cleavage of a pre-obtained substrate (Gly-Pro-aminoluciferin) by DPPIV [20], followed by light production measured as luciferase activity. The luminescent signal recorded for 0.1 seconds, defined as relative light units (RLU), is proportional to the total amount of DPPIV activity in each sample. The assay used 50 µL 0.2% diluted plasma sample and 50 µL freshly prepared CD26/DPPIV-Glo™ reagent followed by an incubation time of 30 minutes at room temperature in accordance with the manufacturer’s protocol, with 50 µL PBS used as negative control. The DPPIV activity is presented as an arbitrary unit (AU) giving the RLU sample/RLU PBS quotient.

### Analysis of IP-10 Concentration

Quantification of IP-10 in the baseline plasma samples was performed using a solid-phase IP-10 ELISA (R&D SYSTEMS, Minneapolis, MN, USA) according to manufacturer’s protocol [33] but with plasma samples diluted 1∶4 using assay diluent.

### Fibrosis Classification

The pretreatment liver biopsies were previously analyzed in a blinded fashion according to the Ishak protocol [14].

### HCV Peptides and Peptide-HLA Class I Tetramers

Synthetic peptides corresponding to different HLA-A2 and HLA-A3 restricted HCV genotype 1a sequences [34] from different nonstructural (NS) HCV proteins were purchased from Chiron Mimotopes: HLA-A2 NS3 CINGVCWTV^1073–1081^, HLA-A2 NS3 KLVALGINAV^1406–1415^, HLA-A3 NS3 LIFCHSKKK^1391–1399^, HLA-A2 NS4 LLFNILGGWV^1807–1816^, HLA-A3 NS4 GVAGALVAFK^1858–1867^, HLA-A3 NS5 RVCEKMALY^2588–2596^, and HLA-A2 NS5 ALYDVVTKL^2594–2602^. Individual tetrameric peptide-HLA class I complexes containing the above peptides were purchased from Proimmune LTD (Oxford, UK).

### Expansion, and Tetramer or Intracellular IFN-γ Staining in Short-term CD8^+^ T cell Lines

Peptide specific polyclonal CD8^+^ T cell lines were generated from frozen PBMCs previously isolated from fresh heparinized blood from 24 genotype 1 and four genotype 2 or 3 patients by Ficoll-Hypaque density gradient centrifugation and resuspended to 3×10^5^/well in RPMI-1640 supplemented with 25 mmol/L HEPES, 2 mmol/L L-glutamine, 50 mg/mL gentamycin, and 10% human serum (complete medium) containing interleukin (IL)-7 (5 ng/mL; Endogen, Woburn, MA) and IL-12 (100 pg/mL; R&D Systems, Abingdon, UK) and stimulated with 1 µmol/L final concentration HLA-A2 or HLA-A3 restricted HCV peptides. Recombinant IL-2 (50 U/mL; EuroCetus, Amsterdam, The Netherlands) was added on day 3 of culture. After 10 days of culture at +37°C with 5% CO_2_, the CD8^+^ T cell lines were washed, stained for tetramer-positive cells, or resuspended to 2×10^6^/mL in complete medium and stimulated with the same peptides at 1 µg/mL at +37°C in 5% CO_2_. As controls, medium or an irrelevant peptide were added. Brefeldin-A (10 µg/ml; Sigma Chemical Co, St. Louis, MO) was added after one hour. After five hours of stimulation, the cells were stained with allophycocyanin-labeled anti-CD8 and peridinin chlorophyll protein-labeled anti-CD3 MoAb, fixed and permeabilized with Cytofix/Cytoperm (BD Bioscience, San Jose, CA) followed by phycoerytrin-labled anti-human IFN-γ (Sigma) labeling. Intracellular IFN-γ expressions in CD8^+^CD3^+^ cells were analyzed on a FACS-Calibur flow cytometer using CELLQuest software (BD). Background IFN-γ-positive CD8^+^ cells values in cells stimulated with controls (range 0%–0.4%) were subtracted from the values of the restimulated cells. CD8^+^ T cells without detectable IFN-γ expression were given a value of 0.009% to be able to be visualized using a log_10_ scale.

### Statistical Analyses

Mann Whitney-U test, Spearman correlation (*r_s_*), Kruskal-Wallis test, χ^2^ test, receiver operating characteristic (ROC) calculating the area under the curve (AUC), and stepwise binary logistic regression analyses were used. The sCD26 concentration cut-off value was determined from the intersection of the sensitivity and specificity obtained in the ROC analysis using a two graph-ROC (TG-ROC) curve [35]. All statistical analyses were performed using Prism (Version 5.0c, GraphPad Software, La Jolla, CA) or SPSS (Version 20.0.0, IBM Corp, Armonk, NY, USA) software. All reported *P*-values are two-sided.

## Results

### sCD26 and Treatment Outcome in Genotype 1–3 Patients from Two Independent Studies

**Figure 1:**
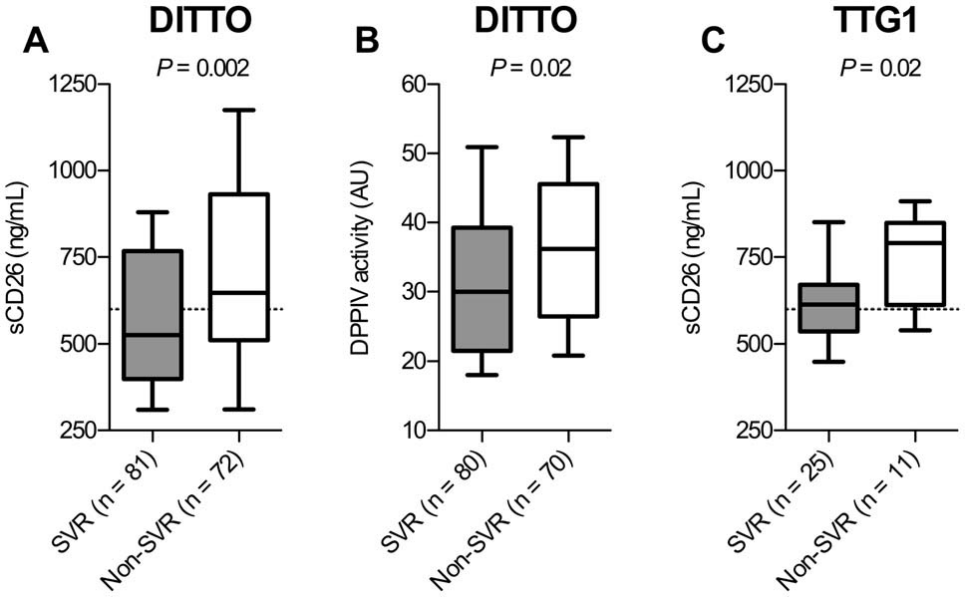
CD26 in genotype 1 patients from the DITTO-HCV and TTG studies grouped depending on treatment outcome. Pretreatment plasma sCD26 concentrations (ng/mL) (**A**) and DPPIV activity (AU) (**B**) in patients from the DITTO-HCV study, and pretreatment plasma sCD26 concentrations (ng/mL) (**C**) in the TTG1 study grouped depending of treatment outcome. Box plots display the 10^th^, 25^th^, 50^th^, 75^th^, and 90^th^ percentiles. The dashed line indicates the 600 ng/mL sCD26 cut-off concentration. Statistics analysis using two-tailed Mann-Whitney U-test.

Eighty-four percent of the genotype 1–3 patients included in the DITTO study were available for sCD26 analysis. Neither the included genotype 1 (Table 1) nor genotype 2/3 (Table 2) patients differed significantly regarding the evaluated parameters compared with the full DITTO study cohort. The included genotype 1 patients from the DITTO study who responded to therapy displayed significantly lower baseline sCD26 concentration (*P = *0.002; Figure 1A) and significantly lower DPPIV activity (*P = *0.02; Figure 1B) compared with patients failing treatment. There was an overall weak, albeit highly significant, correlation between the sCD26 concentration and the DPPIV activity (*r_s_* = 0.35, *P = *0.0001, n = 150).

**Figure 2:**
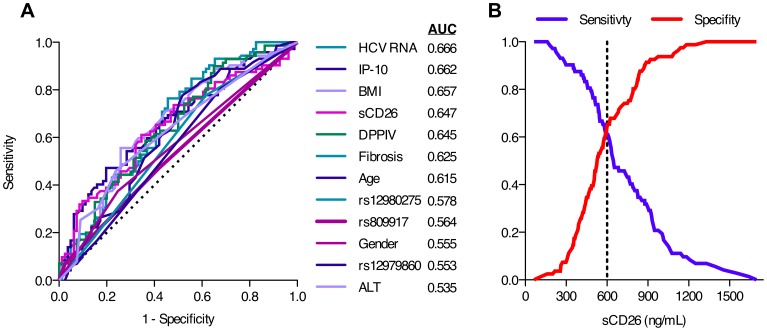
ROC analysis and assessment of the sCD26 concentration cut-off value. (**A**) ROC curve analysis of the indicated baseline factors with the AUC value. A diagonal reference line is shown using a dashed line. (**B**) TG-ROC analysis for determining the cut-off value for the sCD26 concentration show the sensitivity and the specificity from the ROC curve. The dashed line indicates where the sensitivity and specificity intersects.

A ROC analysis was performed to evaluate the baseline sCD26 concentration with regards to previously established predictors of SVR. The baseline levels of HCV RNA (0.666) and IP-10 (0.662) showed the highest AUC values followed by the baseline sCD26 concentration (0.647) and the DPPIV activity (0.645; Figure 2A). The TG-ROC analysis determined the baseline sCD26 concentration cut-off value for the genotype 1 patients in the DITTO study to 600 ng/mL sCD26 by choosing the sCD26 concentration where the sensitivity intersected with the specificity (Figure 2B) [36]. The patients with sCD26 concentrations <600 ng/mL had a significantly greater decline in HCV RNA day 0 to 1 (*P = *0.005) as well as a significant higher likelihood of achieving SVR (*P = *0.01) (Table 3). Patients with lower sCD26 concentrations also showed significantly lower concentrations of IP-10 (*P* = 0.04), as well as lower levels of HCV RNA (*P* = 0.02) and ALT (*P* = 0.03) along with a lower BMI and a higher proportion of female gender (Table 4). However, the sCD26 did not significantly correlate with either ALT (*r_s_* = 0.155, *P = *0.06, n = 153) or HCV RNA (*r_s_* = 0.120, *P = *0.14, n = 153), but correlated weakly albeit significantly with IP-10 (*r_s_* = 0.261, *P = *0.001, n = 150).

**Table 3:** On-treatment responses of the DITTO-HCV genotype 1 patients grouped below or above the sCD26 600 ng/mL cut-off concentration prior to start of therapy.

	<600 ng/mL (n = 81)	>600 ng/mL (n = 72)	*P*
**Log_10_ HCV RNA (IU/mL) decline 1^st^ day on treatment**	0.91 (−0.72–3.07)	0.61 (−1.05–2.3)	0.005^a^
**Log_10_ HCV RNA (IU/mL) decline day 8 to 29 on treatment**	1.60 (−0.40–4.89)	1.18 (−2.94–5.81)	0.06^a^
**RVR**	23 (28%)	12 (17%)	0.09^b^
**SVR**	53 (65%)	28 (39%)	0.01^b^

Median (range) or individuals (percent). Statistics using^ a^Mann-Whitney *U* test and ^b^χ^2^ test.

**Table 4:** Baseline characteristics of the DITTO-HCV genotype 1 patients grouped below or above the sCD26 600 ng/mL cut-off concentration prior to start of therapy.

	<600 ng/mL (n = 81)	>600 ng/mL (n = 72)	*P*
**Female**	31 (38%)	16 (22%)	0.03^a^
**BMI (kg/m^2^)**	24.0 (18.8–35.1)	25.1 (19.1–39.8)	0.02^b^
**Age (years)**	40.0 (22.0–64.0)	43.0 (18.0–64.0)	0.6^b^
**IP-10 (pg/mL)**	208 (29.0–2200)	280 (53.0–2200)	0.04^b^
**IP-10 (<150/150–600/>600 pg/mL)**	26/45/10	16/44/12	0.4^a^
**DPPIV activity (AU)**	28.7 (15.6–66.9)	36.7 (16.7–113)	0.001^b^
**HCV RNA (IU/mL)**	6.13 (2.92–7.17)	6.38 (4.36–7.22)	0.02^b^
***rs12979860*** ** (CC/CT/TT)**	23/46/12	17/42/13	0.7^a^
***rs8099917*** ** (TT/TG/GG)**	45/33/3	40/28/4	0.9^a^
***rs12980275*** ** (AA/AG/GG)**	28/40/13	19/41/12	0.5^a^
**ALT (IU/L)**	73.0 (18.0–287.4)	77.0 (18.0–335.3)	0.03^b^
**Fibrosis Stage (Ishak 0/1/2/3/4/5/6)**	6/24/19/7/5/2/4	4/16/16/8/3/10/6	0.2^a^

Median (range) or individuals (percent). Statistics using ^a^χ^2^ test and ^b^Mann-Whitney *U* test.

In order to further evaluate the predictive value of the baseline sCD26 concentration and the 600 ng/mL sCD26 concentration cut-off for treatment outcome, sCD26 concentrations in 36 patients chronically infected with HCV genotype 1 from an independent study (the TTG1 trial) were assessed. In line with the results from the DITTO-HCV study, patients achieving SVR (n = 25) showed significantly lower sCD26 concentrations compared with patients who did not achieve SVR (n = 11) (*P = *0.02; Figure 1C), however without any significant differences in DPPIV activity (*P = *0.4). The 600 ng/mL sCD26 cut-off value yielded a 93% (12 of 13) SVR rate for the patients below 600 ng/mL compared with a 57% (13 of 23) SVR rate for of the patients above 600 ng/mL sCD26 (*P* = 0.03).

**Figure 3:**
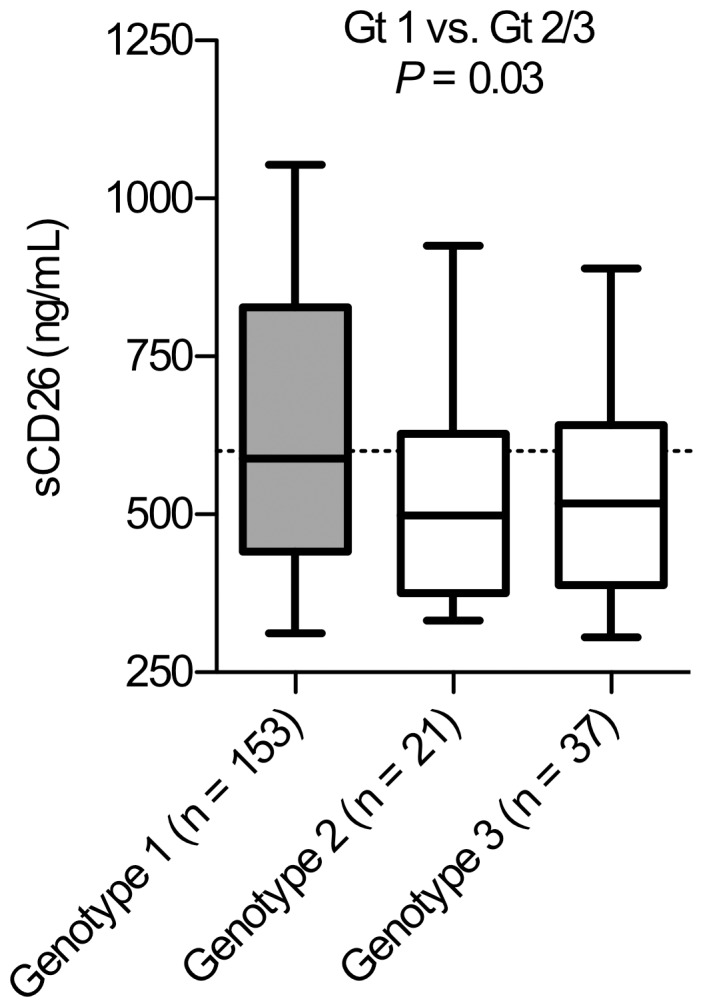
Baseline sCD26 concentrations in genotype 1, 2 and 3 patients in the DITTO-HCV study. Pretreatment plasma sCD26 concentrations (ng/mL) in patients grouped depending on genotype (Gt). Box plots display the 10^th^, 25^th^, 50^th^, 75^th^, and 90^th^ percentiles. The dashed line indicates the 600 ng/mL sCD26 cut-off concentration. Statistics analysis for genotype 1 vs. genotype 2 and 3 using two-tailed Mann-Whitney U-test.

Interestingly, genotype 2 or 3 infected patients showed significantly lower baseline sCD26 concentrations compared with the genotype 1 patients (*P = *0.03; Figure 3) with a trend towards higher sCD26 concentrations for the three genotype 2/3 patients not achieving SVR (median 498 vs. 618 ng/mL sCD26 for SVR and non-SVR patients respectively; n = 58, *P = *0.07). In addition, grouping the patients above or below the 600 ng/mL sCD26 cut-off resulted in 100% (37 of 37) SVR for the patients with <600 ng/mL sCD26 and 86% (18 of 21) SVR for the >600 ng/mL sCD26 patients (*P* = 0.02).

### sCD26 Independently Predicts SVR

In order to determine if sCD26 independently impacts treatment outcome for HCV genotype 1 infected patients, a stepwise binary logistic regression was performed using the baseline factors in Table 4. Lower sCD26 concentrations independently predicted SVR among HCV genotype 1 infected patients, with a 0.2% odds reduction for achieving SVR for each incremental ng/mL sCD26 concentration increase (*P* < 0.05; Table 5). The other independent baseline predictive markers of SVR were lower HCV RNA concentration (*P = *0.001), lower BMI (*P = *0.01), male gender (*P = *0.004), and favorable *IL28B_rs12980275_* genetic variant (*P = *0.03). Furthermore, using the 600 ng/mL sCD26 cut-off concentration resulted in 65% sensitivity, 61% specificity, a 65% positive predictive value (PPV), and a 61% negative predictive value (NPV) (Table 6).

**Table 5:** Odds ratio (OR) and stepwise binary logistic regression analysis identifying pretreatment factors independently predictive of SVR in DITTO-HCV genotype 1 patients.

Parameter	OR	Binary logistic regression analysis (*P*)
**HCV RNA (log_10_ IU/mL)**	0.300 (0.150–0.599)	0.001
**Gender (Male)**	0.265 (0.108–0.650)	0.004
**BMI (kg/m^2^)**	0.867 (0.774–0.970)	0.01
**sCD26 (ng/mL)**	0.999 (0.997–1.000)	0.049
***rs12980275*** ** (AA/AG/GG)**	0.497 (0.270–0.915)	0.03

OR with 95% confidence intervals for the factors analyzed, with values going from low to high or as indicated in the parenthesis.

**Table 6:** Predictive values for SVR among the DITTO-HCV genotype 1 patients included in the study.

Parameter	Sensitivity	Specificity		PPV		NPV
**CC** ***_rs12979860_***	32% (n = 81)		81% (n = 72)		65% (n = 40)	51% (n = 113)
***AA_rs12980275_***	38% (n = 81)		78% (n = 72)		66% (n = 47)	53% (n = 106)
**TT** ***_rs8099917_***	58% (n = 81)		47% (n = 72)		55% (n = 85)	50% (n = 68)
**<150 pg/mL IP-10**	33% (n = 81)		79% (n = 72)		64% (n = 42)	57% (n = 111)
**<600 pg/mL IP-10**	94% (n = 81)		24% (n = 72)		58% (n = 131)	77% (n = 22)
**<600 ng/mL sCD26**	65% (n = 81)		61% (n = 72)		65% (n = 81)	61% (n = 72)
**Gender (Female)**	25% (n = 81)		63% (n = 72)		43% (n = 47)	45% (n = 106)
**<2×10^6^ IU/mL HCV RNA**	63% (n = 81)		54% (n = 72)		61% (n = 84)	57% (n = 69)
**<25 kg/m^2^ BMI**	38% (n = 81)		38% (n = 72)		41% (n = 76)	35% (n = 77)
**<43 years Age**	58% (n = 81)		57% (n = 72)		60% (n = 78)	55% (n = 75)

Sensitivity, specificity, positive predictive value (PPV), and negative predictive value (NPV) for each parameter.

We have previously reported that IP-10 levels are weakly but significantly associated with *IL28B* genetic variants [13]. However, no such association was observed between the baseline sCD26 concentration and *IL28B rs12970860* (*P = *0.4, Kruskal-Wallis test), *rs12980275* (*P = *0.6), or *rs809917* (*P = *0.6) SNPs or *IL28B* genotype distribution (Table 4) for the DITTO-HCV genotype 1 patients. In agreement with the observation that there was no association between the baseline sCD26 concentration and *IL28B* genotypes, having below the 600 ng/mL sCD26 cut-off value significantly improved the treatment response rate in the genotype 1 DITTO-HCV patients with one or two *IL28B* risk alleles (CT/TT*_rs12970860_ P = *0.04, AG/GG*_rs12980275_ P = *0.01, and *rs809917 P = *0.007; Table 7). Similarly, lower sCD26 concentrations significantly increased the likelihood of achieving SVR in patients with baseline plasma IP-10 concentrations between 150 pg/mL and 600 pg/mL (*P = *0.002, Table 7).

**Table 7:** The impact of *IL28B* polymorphisms, baseline plasma IP-10 and sCD26 concentrations on the likelihood of achieving SVR for the DITTO-HCV genotype 1 patients grouped using the *IL28B* genotypes, or the IP-10 or the sCD26 cut-offs.

NCBI dbSNP ID		sCD26 <600 ng/mL	sCD26 >600 ng/mL	*P*
***rs12979860***				
CC		18/23 (78%)	8/17 (53%)	0.01
CT/TT		35/58 (60%)	20/55 (37%)	0.04
***P***		0.1	0.4	
***rs12980275***				
AA		21/28 (75%)	10/19 (53%)	0.1
AG/GG		32/53 (60%)	18/53 (34%)	0.01
***P***		0.2	0.2	
***rs8099917***				
TT		29/45 (64%)	18/40 (45%)	0.08
TG/GG		24/36 (67%)	10/32 (31%)	0.007
***P***		0.8	0.2	
**IP-10**				
<150 pg/mL		18/26 (69%)	9/16 (56%)	0.4
150–600 pg/mL		32/45 (71%)	17/44 (39%)	0.002
>600 pg/mL		3/10 (30%)	2/12 (17%)	0.5
***P***		0.04	0.1	

*P*-values using χ^2^ test.

### Short-term HCV-specific CD8^+^ T cells

**Figure 4:**
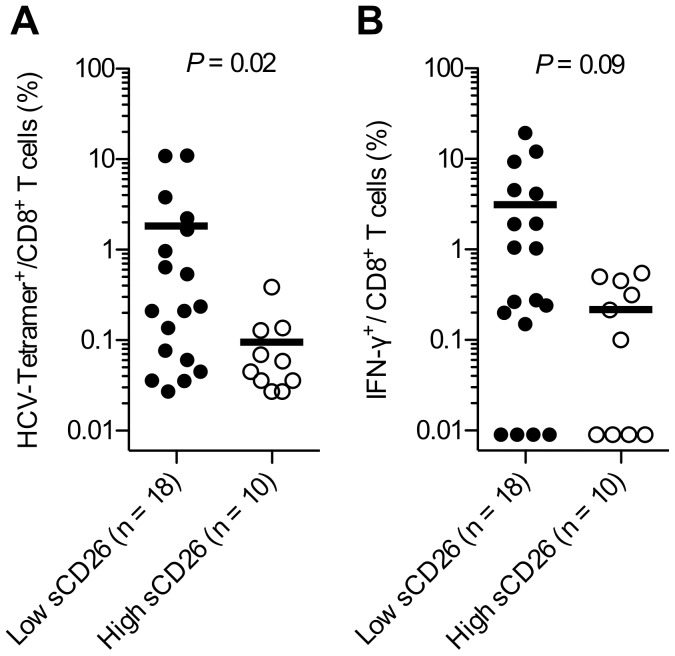
IFN-γ^+^ CD8^+^ T cells after stimulation with genotype 1a HCV specific HLA-A2 or HLA-A3 restricted peptides after *in vitro* expansion. (**A**) Frequency of HCV-tetramer^+^ CD8^+^ T cells after 10 days *in vitro* expansion for patients (n = 28) grouped above or below the median for sCD26. (**B**) Frequency of IFN-γ^+^ CD8^+^ T cells after 10 days *in vitro* expansion for patients (n = 28) grouped above or below the median for sCD26. Statistic using two-tailed Mann-Whitney U-test.

To evaluate the association between sCD26 concentrations and the HCV-specific CD8^+^ T cell response, PBMCs collected prior to therapy from 28 HLA-A2 or HLA-A3 positive patients were analyzed for their ability to recognize and produce IFN-γ after stimulation with HLA-A2 or HLA-A3 restricted genotype 1a peptides. When grouping the patients above or below 600 ng/mL sCD26, it was observed that patients below the cut-off had significantly more HCV-tetramer^+^ CD8^+^ T cells (*P = *0.02, Figure 4A) with a similar trend towards more IFN-γ producing CD8^+^ T cells following stimulation with HCV specific peptides (*P = *0.09, Figure 4B) compared with patients with sCD26 above the cut-off concentration prior to therapy. In line with this observation, a negative correlation between the sCD26 level and the percent HCV-tetramer^+^ CD8^+^ T cells was observed (*r_s = _*−0.41, *P = *0.03, n = 28). Furthermore, a strong correlation was observed between the percentage of HCV-tetramer^+^ CD8^+^ T cells and the percentage of IFN-γ producing CD8^+^ T cells among patients below the sCD26 cut-off (*r_s_ = *0.87, *P = *0.0001, n = 18). However, no such correlation was observed among patients above the sCD26 cut-off (*r_s_ = *−0.11, *P = *0.8, n = 10).

Additionally, no associations were noted between the frequencies of HCV-tetramer^+^ CD8^+^ T cells (*P = *0.4) or IFN-γ^+^ CD8^+^ T cells (*P = *0.7) when grouping the patients above or below the baseline median IP-10 concentration, or between the frequencies of HCV-tetramer^+^ CD8^+^ T cells (*P = *0.2) or IFN-γ^+^ CD8^+^ T cells (*P = *0.5) and the *IL28B_rs12979860_* CC versus non-CC genotypes.

## Discussion

The main finding in the present study was that genotype 1 infected patients achieving SVR after treatment with pegIFN-α/ribavirin demonstrated lower baseline plasma sCD26 concentrations in two independent studies. Lower sCD26 concentrations were not associated with *IL28B* genetic variation, and consequently having lower baseline sCD26 concentrations significantly improved the likelihood of achieving SVR among all *IL28B* SNP risk alleles in HCV genotype 1 infected patients.

Additionally in these two studies, the sCD26 concentration was a more reliable predictor of treatment outcome than DPPIV activity. The surprisingly weak, but highly significant, correlation between sCD26 concentration and DPPIV activity could in part be explained by the existence of both cleaved membrane bound and soluble CD26 in plasma with inherently different DPPIV activity [37], or by differential impact of storage time or possible freeze-thawing. It would have been of interest to measure the impact of IP-10 truncation on SVR in the current studies, however the levels of truncated IP-10 could not be quantified subsequent to the lack of DPPIV inhibitors at the time of sampling, which is necessary to prevent artifactual extravascular IP-10 truncation [20]. Also, having female gender is usually a positive predictor of SVR following HCV therapy, however, the complete DITTO study has previously shown a higher SVR rate among male patients [14] and accordingly the DITTO cohort in the present study also demonstrated male gender to be associated with favorable outcome (Table 5). CD26 has previously been suggested to be a marker for liver disease [38] and higher sCD26 could thus be an indicator of liver injury. In the present study however, the distribution of fibrosis stages were not significantly different when grouping the patients above or below the sCD26 cut-off (Table 4). Also the sCD26 concentration did not significantly correlate with ALT, and the genotype 2/3 cohort showed higher ALT despite having lower sCD26 concentrations as compared with genotype 1 infected patients (Table 1 and 2).

Interestingly, it was observed that the sCD26 concentrations were significantly lower among HCV genotype 2 and 3 infected patients than among those infected with genotype 1. A similar non-significant trend towards lower baseline IP-10 levels for HCV genotype 2/3 in comparison with genotype 1/4 infected patients has previously been reported from the same study cohort [14], [15]. The mechanisms underlying these observations merit further investigation.

Previous studies have correlated exhausted HCV-specific CD8^+^ T cells with treatment failure [26]. To evaluate whether the sCD26 concentration could function as a marker of functional HCV-specific T cells, short-term HCV-specific CD8^+^ T cells from HLA-A2^+^ or HLA-A3^+^ patients were generated along with assessment of the ability of these cells to produce IFN-γ after stimulation with HCV specific peptides. Interestingly, these data suggested, albeit obtained from a small number of patients (n = 28), that a low baseline sCD26 concentration may be associated with the presence of functional HCV-specific T cells, whereas higher sCD26 concentration could indicate a T cell exhausted phenotype that has previously been described in patients with chronic HCV infection who fail therapy [39]. The limited number of patients available for T cell analyzes only differed significantly compared with the complete cohort with regards to baseline IP-10 for the four genotype 2/3 infected patients (Table 1 and 2), thus suggesting the evaluated patients may be representative of the larger cohort. Treatment response mechanisms, however, are complex, and the presence of functional HCV-specific T cells does not consistently entail improved likelihood of achieving SVR [28].

In conclusion and in agreement with the report by Casrouge *et al.* (2011) [20], the present study suggests that lower baseline sCD26 concentrations are associated with improved response to combination therapy for HCV genotype 1 infection. Furthermore, we could show that having sCD26 concentrations below the 600 ng/mL sCD26 cut-off value augmented the predictive value of both baseline IP-10 concentration and *IL28B* genetic variants. The very rapid clearance of HCV viremia observed following the recent introduction of new DAAs for HCV, including nucleotide polymerase inhibitors, likely will hamper the utility of on-treatment levels of HCV RNA in tailoring therapy. Thus it is reasonable to assume that baseline markers of response, such as sCD26 concentration, may increase in importance in order to personalize HCV treatment duration or choice of therapy as well as reduce cost. However, further prospective studies to validate the sCD26 concentration and its association with HCV-specific T cells are warranted.
